# Effects of air pollution on neonatal prematurity in guangzhou of china: a time-series study

**DOI:** 10.1186/1476-069X-10-2

**Published:** 2011-01-10

**Authors:** Qingguo Zhao, Zhijiang Liang, Shijuan Tao, Juan Zhu, Yukai Du

**Affiliations:** 1 College of Public Health, Tongji Medical College, Huazhong University of Science and Technology, Wuhan 430030, Hubei, PR China; 2 Guangdong Women and Children Health Hospital, Guangzhou 510010, Guangdong, PR China

## Abstract

**Background:**

Over the last decade, a few studies have investigated the possible adverse effects of ambient air pollution on preterm birth. However, the correlation between them still remains unclear, due to insufficient evidences.

**Methods:**

The correlation between air pollution and preterm birth in Guangzhou city was examined by using the Generalized Additive Model (GAM) extended Poisson regression model in which we controlled the confounding factors such as meteorological factors, time trends, weather and day of the week (DOW). We also adjusted the co linearity of air pollutants by using Principal Component Analysis. The meteorological data and air pollution data were obtained from the Meteorological Bureau and the Environmental Monitoring Centre, while the medical records of newborns were collected from the perinatal health database of all obstetric institutions in Guangzhou, China in 2007.

**Results:**

In 2007, the average daily concentrations of NO_2_, PM_10 _and SO_2 _in Guangzhou, were 61.04, 82.51 and 51.67 μg/m^3 ^respectively, where each day an average of 21.47 preterm babies were delivered. Pearson correlation analysis suggested a negative correlation between the concentrations of NO_2_, PM_10_, SO_2, _and temperature as well as relative humidity. As for the time-series GAM analysis, the results of single air pollutant model suggested that the cumulative effects of NO_2_, PM_10 _and SO_2 _reached its peak on day 3, day 4 and day 3 respectively. An increase of 100 μg/m^3 ^of air pollutants corresponded to relative risks (RRs) of 1.0542 (95%CI: 1.0080 ~1.1003), 1.0688 (95%CI: 1.0074 ~1.1301) and 1.1298 (95%CI: 1.0480 ~1.2116) respectively. After adjusting co linearity by using the Principal Component Analysis, the GAM model of the three air pollutants suggested that an increase of 100 μg/m^3 ^of air pollutants corresponded to RRs of 1.0185 (95%CI: 1.0056~1.0313), 1.0215 (95%CI: 1.0066 ~1.0365) and 1.0326 (95%CI: 1.0101 ~1.0552) on day 0; and RRs of the three air pollutants, at their strongest cumulative effects, were 1.0219 (95%CI: 1.0053~1.0386), 1.0274 (95%CI: 1.0066~1.0482) and 1.0388 (95%CI: 1.0096 ~1.0681) respectively.

**Conclusions:**

This study indicates that the daily concentrations of air pollutants such as NO_2_, PM_10 _and SO_2 _have a positive correlation with the preterm births in Guangzhou, China.

## Background

Air pollution affects the health of children as well as the elderly, and it has been increasingly noticed and also studied in the recent years as a new public health challenge. Some studies have already shown that air pollution is associated with an increased risk rate of adverse pregnancy outcomes [1,2]. International survey data showed a 7-10% premature rate [3] in the industrialized countries, and 9-12% in United States in recent years, displaying an upward trend [4]. A survey in China indicated a 5-15% preterm rate increasing [5].

Fifteen percent of preterm babies die in neonatal period. In addition to fatal malformations, 70% of neonatal deaths and 75% of neonatal complications are associated with premature births [6]. Complications include respiratory diseases, intracerebral hemorrhage, infections and dysplasia. Compared with full-term babies, premature babies suffer greater exposure to cerebral palsy, amblyopia, deafness and mental retardation [7]. It has been shown that prematurity was not only a major cause in neonatal deaths, but also a substantial contributor to diabetes mellitus, coronary heart disease and hypertension in adulthood [8,9]. Hence, seeking the causes and the risk factors of preterm birth is of vital importance to public health.

By the end of the twentieth century, researches on possible risk factors of premature birth were mainly focused on the socio-economics level, educational achievements, smoking status and drinking behavior during pregnancy, intrauterine infections, multiple births, parity history including abortions, still births and preterm births, genital abnormality, pregnancy-induced hypertension, risky sexual behavior, etc [10-15]. During the last decade, an increasing number of researchers have noticed the possible causality between air pollutants and the occurrence of prematurity, which lead to a large number of studies in America, Canada, Australia, Lithuania and China concerning this topic. The results suggested that exposure to air pollutants such as NO_2_, PM_10 _and SO_2 _during pregnancy were possibly related to premature births [16-19]. Meanwhile, cities in China such as Beijing, Taiyuan and Taibei have also conducted such studies revealing that the increased concentration of air pollutants such as NO_2_, PM_10 _and SO_2 _presents as a risk attributing to premature births [20-22]. In this study we applied the Generalized Additive Model (GAM) extended Poisson regression model to quantitatively evaluate the effects of ambient air pollutants, NO_2_, PM_10 _and SO_2_, on the preterm birth by analyzing the time-series data of air pollution, meteorological factors, and preterm births in Guangdong Province in 2007.

## Methods

### Data collection

Guangzhou, lied in the south of China, composed of ten districts and two satellite cities, has an urban area of over 7,434,400,000 m^2 ^and a metropolitan area population of 9.8 million. We obtained the information of all the live births in Guangzhou in 2007, by using the birth registry database which covers all the obstetric clinics in Guangzhou. There are a total of 142,312 births in Guangzhou City from 2007 January 1^st ^till 2007 December 31^st^, including 9,083 (6.38%) preterm births. Gestational age was computed as the number of weeks between the date of the last menstrual period (LMP) and the date of birth [23]. Eligible births with gestational ages <37 weeks were considered preterm. Twin pregnancy and multiple pregnancies were excluded from this study. After exclusions, 7,836 of 9,083 preterm births (86.27%) were defined as the analytic units of the study. The number of preterm births was tallied for each day in 2007.

The data for the daily mean concentrations of air pollutants nitrogen dioxide (NO_2_), particulate matter less than or equal to 10 microns (PM_10_) and sulfur dioxide (SO_2_) in 2007 were collected from the Environmental Monitoring Center of Guangzhou city. The daily concentrations of each pollutant were averaged from the available monitoring results of nine fixed-site stations located in the urban areas of Guangzhou, which were monitored by the China National Quality Control. We collected the 24-hour average concentrations for PM_10_, SO_2 _and NO_2 _by applying a selection criterion that at least 75% of all the one-hour values in a given day are available.

To allow the adjustment for the possible influences of weather on preterm birth, daily average temperature (°C) and relative humidity (%) data were collected from Guangzhou Meteorological Bureau. The weather data were measured at a fix-site station located in Yuexiu District of Guangzhou.

### Statistical analysis

Given the total population, daily premature birth is relatively an event with small probabilities on the demographical scale. As a typical time-series data, its distribution approximately follows the Poission distribution [16]. To determine the influence of air pollution on premature birth, analysis should be carried out in time-series Generalized Additive Model (GAM) extended Poisson regression [24], which expands the traditional Generalized Log-Linear Model. In addition to fitting common linear subjects, complicated non-linear variables of induced variables were incorporated in different functions of additive operations. The non-parametric flexibility of GAMs has resulted in their widespread use in time-series studies to adjust for the nonlinear confounding effects of seasonality and trend [25-32]. Since introduced by Schwartz J in 1996 [33], time-series Generalized Additive Model (GAM) extended Poisson regression has become a standard method to conduct air pollution researches in environmental epidemiology. The formula is explained in detail as follows:

log[E(Yt)]=α+βZt+S(time,df)+S(temperature,df)+S(relative humidity,df)+DOW(day of week)

In the formula, Yt--t-represents daily number of preterm babies, E(Yt)--t-expected value of daily number of preterm babies, α--residual, β--regression coefficient, Zt--t-concentration of air pollution or accumulated average concentration over several days, S(time, df)--calendar time smoothing spline function, S(temperature, df)--temperature smoothing spline function, S(relative humidity, df)--relative humidity smoothing spline function, DOW(day of week)--dummy variable.

In this study, we firstly built basic models on the daily numbers of preterm births without analyzing the air pollution variables. We approached with the smoothing spline functions while incorporating time-independent variables, including calendar time, temperature and relative humidity, to control for the nonlinear confounding effects of trend, seasonality and weather [34]. This can accommodate non-linear and non-monotonic patterns between preterm birth and time/weather conditions, and thus we created a flexible modeling tool [35]. Meanwhile, dummy variable was also used to control the effects of "day of the week" (DOW). Residuals of each model were examined to check whether there were discernible patterns and autocorrelation by means of residual plots and partial autocorrelation function plots, respectively [23].

After the establishment of basic models, we added the pollutant variables into the models and analyzed their effects on preterm births. The number of gestations at risk for preterm birth was used as an offset. Generalized cross-validation (GCV) scores were used to compare the relative quality of the incidence of preterm predictions across these non-nested models and verify how well the models fit the data [35]. Taking into considerations the delayed effects of air pollutants, the model also included lag effects. The purpose of the study was to investigate the effects of air pollutants on health over a short-time period, and we applied the criteria of including the days before 7 lag day, based on literature review [36,37]. Lag day is specifically used in time-series analysis. We compared the health index of day 0 and previous days, by analyzing the different level of air pollution concentration, and also used this model to predict the health impacts on the future [38]. We introduced the concentration of air pollutants on day 0, one day ago, seven days ago (Lag0-Lag7) or lag moving average (Avg0-Avg7) into the model one by one to calculate the relative risk and CI by the regression coefficient β of air pollutants to make it possible to quantify the influence of air pollutants on premature birth. Moreover, sensitivity analysis was also conducted within the established models. While carrying out the sensitivity test, multiple air pollutants model was fitted to evaluate the stability of single air pollutant model and to compare lag and cumulative effects to analyze the stability of air pollutants' effects.

We also introduced "Principal Component" into the study and we established the dose-response model of multiple ambient air pollutants' health effects in order to exclude the impacts of co linearity [39]. The composite latent variable (Principal Component) suitable for variable information of original air pollutants by principal component analysis was substituted into the time-series Generalized Additive Model (GAM) extended Poisson regression model. The variables were also fitted into the linear model of principal components. Meanwhile, we transformed the regression coefficient β of the principal components into the regression coefficient b of the original air pollutants, and at last we calculated the relative risk and CI so as to quantify the influence of each air pollutant on preterm birth in the multiple air pollutants model.

All the above statistical analyses were conducted by using R 2.9.0.

## Results

### Descriptive statistical results of premature births

**Figure 1:**
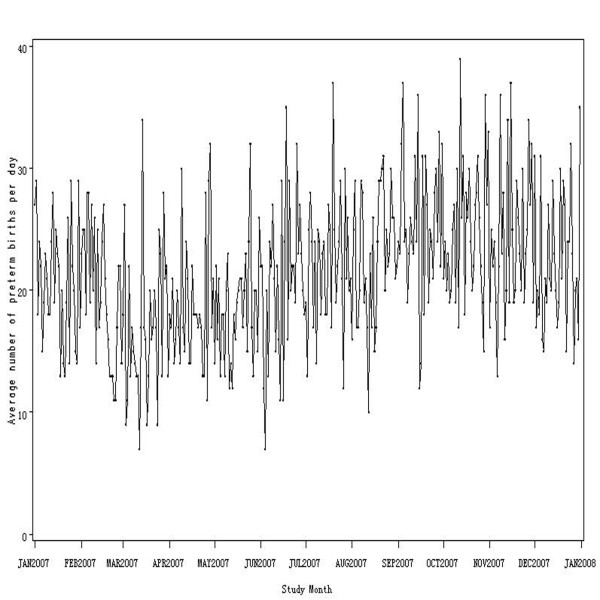
**Average number of preterm births per day by study month in Guangzhou, China in 2007**.

Figure 1 shows the variations in preterm births over the entire study period. The average of preterm birth was 21.47 every day in 2007, the quartile range was 8, P_0_, P_25_, P_50_, P_75_, P_100 _was 7.00, 17.00, 21.00, 25.00, 39.00 respectively.

### Descriptive statistical results of air pollutants and meteorological factors

Table 1 shows the daily average concentrations of NO_2, _PM_10 _and SO_2 _in Guangzhou were 61.04 μg/m^3^, 82.51 μg/m^3^, 51.67 μg/m^3 ^respectively, while daily average temperature in 2007 was (23.87±5.76)°C (n = 365), P_0_, P_25_, P_50_, P_75_, P_100 _corresponds to 8.00, 19.60, 24.50, 28.77, 32.65°C, the quartile range was 9.17°C, relative humidity was (67.55 ± 10.26)%(n = 365), P_0_, P_25_, P_50_, P_75_, P_100 _were 40.00%, 62.50%, 67.50%, 75.00%, 88.00% respectively. The quartile range was 12.50%.

**Table 1:** Meteorological and ambient air pollution index (n = 365) in Guangzhou, China in 2007

Index	Mean	SD	P_0_	P_25_	P_50_	P_75_	P_100_	P_75_-P_25_
Temperature (°C)	23.87	5.76	8.00	19.60	24.50	28.77	32.65	9.17

Relative humidity (%)	67.55	10.26	40.00	62.50	67.50	75.00	88.00	12.50

NO_2_(μg/m^3^)	61.04	63.80	15.22	26.55	38.11	59.89	467.89	33.33

PM_10_(μg/m^3^)	82.51	52.71	19.67	49.22	69.78	94.44	405.28	45.22

SO_2_(μg/m^3^)	51.67	34.84	8.67	28.89	43.67	63.44	194.94	34.56

### Correlation analysis of ambient air pollution and Meteorological factors indexes

Pearson correlation analysis in Table 2 indicated that temperature and relative humidity were negatively associated with all three air pollutants. The absolute r value of NO_2_, PM_10 _and SO_2 _to temperature was 0.2799, 0.1868 and 0.1650 respectively, and to relative humidity, 0.2126, 0.2002 and 0.0577. The results suggested that with a decrease in temperature and relative humidity, the concentration of air pollutants would increase. The correlation of three air pollutants indicated a strong statistical significance (P < 0.01) with the strongest correlation being between NO_2 _and PM_10 _(r value is 0.8533), and then between NO_2 _and SO_2 _(r value is 0.8440). As for temperature and relative humidity, the r value was 0.1924 and statistically significant (P < 0.01). Correlation analysis of the above indexes showed possible co linearity for the independent variables.

**Table 2:** Pearson correlation coefficient of meteorological and air pollutants

Index	Temperature	Relative humidity	NO_2_	PM_10_	SO_2_
Temperature	1.0000	0.1924**	-0.2799**	-0.1868**	-0.1650**

Relative humidity		1.0000	-0.2126**	-0.2002**	-0.0577

NO_2_			1.0000	0.8533**	0.8440**

PM_10_				1.0000	0.7489**

SO_2_					1.0000

Notes: *P < 0.05, **P < 0.01

### Lag effects and cumulative effects using a single air pollutant model

Starting with the single air pollutant model and aiming to define the strongest time concentration of health effect, analytical results of the lag effect (Table 3, 4 and 5) showed that NO_2_, PM_10 _and SO_2 _had statistical significance (P < 0.05) only on day 0; analytical results of the cumulative effects (Table 3, 4 and 5) showed that NO_2 _and SO_2 _had their strongest cumulative effects on day 3 while PM_10 _on day 4, all with statistical significance (P < 0.05).

**Table 3:** Results of single air pollutant model lag effects and cumulative effects of NO_2_

NO_2_	Estimate^(1)^	Std.Error^(2)^	RR	95%CI	t Value	Pr > |t|
Lag effects						

Lag0	0.0416	0.0182	1.0425	1.0068 ~1.0781	2.29	0.0227

Lag1	0.0122	0.0186	1.0123	0.9758 ~1.0488	0.66	0.5119

Lag2	0.0263	0.0186	1.0266	0.9902 ~1.0630	1.42	0.1579

Lag3	0.0364	0.0185	1.0370	1.0007 ~1.0734	1.96	0.0508

Lag4	0.0003	0.0189	1.0003	0.9632 ~1.0375	0.02	0.9865

Lag5	-0.0042	0.0190	0.9959	0.9585 ~1.0332	-0.22	0.8273

Lag6	0.0178	0.0188	1.0179	0.9811 ~1.0548	0.95	0.3451

Lag7	0.0007	0.0191	1.0007	0.9634 ~1.0381	0.04	0.9687

Cumulative effect						

Avg0	0.0416	0.0182	1.0425	1.0068 ~1.0781	2.29	0.0227

Avg1	0.0323	0.0200	1.0328	0.9936 ~1.0721	1.61	0.1075

Avg2	0.0390	0.0219	1.0397	0.9969 ~1.0826	1.78	0.0754

Avg3	0.0527	0.0236	1.0542	1.0080 ~1.1003	2.24	0.0257

Avg4	0.0493	0.0252	1.0506	1.0013 ~1.0999	1.96	0.0506

Avg5	0.0431	0.0265	1.0440	0.9921 ~1.0960	1.63	0.1051

Avg6	0.0461	0.0276	1.0472	0.9931 ~1.1013	1.67	0.0955

Avg7	0.0435	0.0287	1.0445	0.9881 ~1.1008	1.51	0.1310

Notes: (1) Estimate (× 100), (2) Std. Error (× 100)

**Table 4:** Results of single air pollutant model lag effects and cumulative effects of PM_10_

PM_10_	Estimate^(1)^	Std.Error^(2)^	RR	95%CI	t Value	Pr > |t|
Lag effects						

Lag0	0.0500	0.0217	1.0512	1.0087 ~1.0938	2.30	0.0220

Lag1	0.0157	0.0221	1.0158	0.9725 ~1.0592	0.71	0.4771

Lag2	0.0211	0.0222	1.0213	0.9777 ~1.0648	0.95	0.3439

Lag3	0.0422	0.0221	1.0431	0.9997 ~1.0865	1.91	0.0575

Lag4	0.0097	0.0225	1.0097	0.9657 ~1.0538	0.43	0.6663

Lag5	-0.0031	0.0226	0.9969	0.9526 ~1.0413	-0.14	0.8923

Lag6	0.0014	0.0227	1.0014	0.9569 ~1.0458	0.06	0.9525

Lag7	-0.0094	0.0229	0.9906	0.9457 ~1.0355	-0.41	0.6811

Cumulative effect						

Avg0	0.0500	0.0217	1.0512	1.0087 ~1.0938	2.30	0.0220

Avg1	0.0423	0.0243	1.0432	0.9955 ~1.0909	1.74	0.0831

Avg2	0.0486	0.0269	1.0498	0.9972 ~1.1025	1.81	0.0710

Avg3	0.0651	0.0291	1.0673	1.0103 ~1.1243	2.24	0.0258

Avg4	0.0665	0.0313	1.0688	1.0074 ~1.1301	2.13	0.0342

Avg5	0.0608	0.0334	1.0627	0.9972 ~1.1281	1.82	0.0696

Avg6	0.0570	0.0353	1.0586	0.9896 ~1.1277	1.62	0.1069

Avg7	0.0497	0.0371	1.0510	0.9783 ~1.1237	1.34	0.1811

Notes: (1) Estimate (× 100), (2) Std. Error (× 100)

**Table 5:** Results of single air pollutant model lag effects and cumulative effects of SO_2_

SO_2_	Estimate^(1)^	Std.Error^(2)^	RR	95%CI	t Value	Pr > |t|
Lag effects						

Lag0	0.1060	0.0326	1.1118	1.0479 ~1.1757	3.26	0.0012

Lag1	0.0323	0.0331	1.0328	0.9678 ~1.0978	0.97	0.3309

Lag2	0.0432	0.0332	1.0442	0.9791 ~1.1093	1.30	0.1940

Lag3	0.0505	0.0332	1.0518	0.9867 ~1.1168	1.52	0.1291

Lag4	-0.0028	0.0336	0.9972	0.9314 ~1.0631	-0.08	0.9346

Lag5	-0.0154	0.0337	0.9847	0.9186 ~1.0507	-0.46	0.6475

Lag6	-0.0060	0.0337	0.9940	0.9280 ~1.0601	-0.18	0.8591

Lag7	-0.0238	0.0339	0.9765	0.9100 ~1.0429	-0.70	0.4828

Cumulative effect						

Avg0	0.1060	0.0326	1.1118	1.0479 ~1.1757	3.26	0.0012

Avg1	0.0918	0.0358	1.0962	1.0259 ~1.1664	2.56	0.0108

Avg2	0.1020	0.0389	1.1074	1.0312 ~1.1836	2.62	0.0092

Avg3	0.1220	0.0417	1.1298	1.0480 ~1.2116	2.92	0.0037

Avg4	0.1100	0.0443	1.1163	1.0294 ~1.2032	2.49	0.0134

Avg5	0.0912	0.0467	1.0954	1.0039 ~1.1870	1.95	0.0518

Avg6	0.0826	0.0488	1.0861	0.9904 ~1.1818	1.69	0.0916

Avg7	0.0671	0.0510	1.0694	0.9694 ~1.1694	1.32	0.1894

Notes: (1) Estimate (×100), (2) Std. Error (×100)

After comparing the results of lag effects and cumulative effects, we found that lag effects did not last for a long time with only day 0 resulting in statistical significance (P < 0.05). In regard to cumulative effects, the time spans of NO_2 _and PM_10 _were consistent each other appearing on day 0, day 3 and day 4; the strongest effect of NO_2 _was on day 3 while the strongest effect of PM_10 _was on day 4, and they all have statistical significance (P < 0.05). When it came to the cumulative effects of SO_2_, the effects were maintained from day 0 to day 4 with the maximum effect on day 3 (P < 0.05).

### Model sensitivity analysis

While comparing the models of multiple and single air pollutants, the point estimate for air pollutants' effects generally decreased and showed no statistical significance, which was probably related to the strong co linearity between different air pollutants, or due to the fact that multiple air pollutants model would increase the standard error for model fitting, and lead to lower statistical significance.

After comparing the results of lag effects and cumulative effects, it was shown that lag effect could only last for a short time. In other words, effects could only maintain on day 0, while the time span for cumulative effects lasted longer with an increasing trend of point estimate of risk, reaching its maximum on day 3 approximately, indicating that cumulative effects model was more sensitive than that of lag effects.

### Principal component time-series GAM analysis of multiple air pollutants

In order to exclude the co linearity between different air pollutants in multiple model, concentration of multiple air pollutants on day 0 and on the day with the strongest cumulative effects were analyzed by GAM model after being adjusted by principal component, and we also compared the results before and after adjusting.

2.5.1 Principal component time-series GAM analysis of effects on day 0 using multiple air pollutants model

We could see from Table 6 that the eigenvalue of the first principal component was 2.6317 (> 1), providing 87.72% composite information; coefficients of different air pollutants were all positive values and close to each other. Therefore, it was considered that the first principal component constituted the composite index to reflect air pollution. The linear model was fitted by adding it into the GAM model.

**Table 6:** Correlation matrix eigenvalues of multiple air pollutants model on day 0 and corresponding eigenvector

**Principal component**	**Correlation matrix eigenvalue**				**Corresponding eigenvector**		
		
	**Eigenvalue**	**Difference of two eigenvalue**	**Contribution rate**	**Accumulated** **Contribution rate**	**NO_2_(0)**	**PM_10_(0)**	**SO_2_(0)**
	
Z1	2.6317	2.3804	0.8772	0.8772	0.5925	0.5708	0.5684
Z2	0.2513	0.1344	0.0838	0.9610	-0.0310	-0.6890	0.7241
Z3	0.1170	-	0.0390	1.0000	-0.8050	0.4467	0.3906

Table 7 displayed the GAM model results for the effects on day 0 of different air pollutants combinations. After adjusting the collinearity within air pollutants index by principal component analysis, double and triple air pollutants models of NO_2 _and PM_10 _had a transformation from non-statistical to statistical significance (P < 0.05)

**Table 7:** Before- and after-adjusting co linearity by principal component analysis of different air pollutants GAM model combination on day 0

Model	Type correction	NO_2_(Lag0)		PM_10_(Lag0)		SO_2_(Lag0)	
		RR value	95%CI	RR value	95%CI	RR Value	95%CI
NO_2_+ PM_10_	before correction	1.0173	0.9502~1.0844	1.0313	0.9511 ~1.1114	-	-
	
	After correction	1.0229*	1.0045~1.0413	1.0278*	1.0055 ~1.0501	-	-

NO_2_+ SO_2_	before correction	0.9691	0.8976~1.0405	-	-	1.1653*	1.0377 ~1.2929
	
	After correction	1.0270*	1.0088~1.0453	-	-	1.0501*	1.0167 ~1.0835

PM_10_+ SO_2_	before correction	-	-	0.9970	0.9299 ~1.0641	1.1118*	1.0116 ~1.2120
	
	After correction	-	-	1.0349*	1.0124 ~1.0574	1.0533*	1.0192 ~1.0874

Three Pollutants	before correction	0.9592	0.8732~1.0452	1.0182	0.9373 ~1.0991	1.1607*	1.0317 ~1.2896
	
	After correction	1.0185*	1.0056~1.0313	1.0215*	1.0066 ~1.0365	1.0326*	1.0101 ~1.0552

Note: * P < 0.05

In the triple model, after the adjustment by principal component analysis, the relative risk of NO_2 _influencing preterm birth was 1.0185 (95%CI: 1.0056 ~1.0313), PM10 was 1.0215 (95%CI: 1.0066 ~1.0365), SO_2 _was 1.0326 (95%CI: 1.0101 ~1.0552).

2.5.2 Principal component time-series GAM analysis of the strongest cumulative effect using multiple air pollutants model

We could see from Table 8 that the eigenvalue of the first principal component was 2.6592 (> 1), providing 88.64% composite information, and coefficients of different air pollutants were all positive value and close to each other; Therefore it is considered that the first principal component constituted the composite index to reflect air pollution. The linear model was fitted by adding it into the GAM model.

**Table 8:** Correlation matrix Eigen value and its corresponding eigenvector of the strongest cumulative effects for multiple air pollutants model

**Principal component**	**Eigenvalue of correlation matrix**				**Corresponding eigenvector**		
		
	**Eigenvalue**	**Difference of two eigenvalue**	**Contribution rate**	**Accumulated Contribution rate**	**NO_2_(3)**	**PM_10_(4)**	**SO_2_(3)**
	
Z1	2.6592	2.4081	0.8864	0.8864	0.5952	0.5680	0.5684
Z2	0.2510	0.1612	0.0837	0.9701	-0.0042	0.7095	-0.7047
Z3	0.0898	-	0.0299	1.0000	-0.8036	0.4171	0.4246

We could see from Table 9 that the strongest cumulative effects of different air pollutant combinations in the GAM model, only after adjustment of co linearity of the air pollutant indexes by principal component analysis indicate a transformation from non-statistical to statistical significance for both double model and triple model.

**Table 9:** Before and after adjusting co linearity by Principal component analysis for strongest cumulative effect of different air pollutants GAM model combination

Model	Type correction	NO_2_(Avg3)		PM_10_(Avg4)		SO_2_(Avg3)	
		RR value	95%CI	RR Value	95%CI	RR value	95%CI
NO_2_+ PM_10_	Before correction	1.0340	0.9456~1.1224	1.0298	0.9130~1.1466	-	-
	
	after correction	1.0284*	1.0042~1.0527	1.0373*	1.0056~1.0690	-	-

NO_2_+ SO_2_	Before correction	0.9782	0.8718~1.0847	-	-	1.1723	0.9839 ~1.3608
	
	after correction	1.0321*	1.0089~1.0553	-	-	1.0598*	1.0171 ~1.1024

PM_10_+ SO_2_	Before correction	-	-	1.0071	0.9085~1.1058	1.1185	0.9865 ~1.2506
	
	after correction	-	-	1.0441*	1.0125~1.0758	1.0627*	1.0181 ~1.1073

Three pollutants	Before correction	0.9639	0.8373~1.0904	1.0244	0.9072~1.1415	1.1688	0.9797 ~1.3579
	
	after correction	1.0219*	1.0053~1.0386	1.0274*	1.0066~1.0482	1.0388*	1.0096 ~1.0681

Note: * P < 0.05

In triple model, after adjusted by principal component analysis, relative risk for NO_2 _on influencing preterm birth was suggested to be 1.0219 (95%CI: 1.0053~1.0386), PM_10 _was 1.0274 (95%CI: 1.0066~1.0482), SO_2 _was 1.0388 (95%CI: 1.0096~1.0681).

## Discussion

The results of the study have shown that the concentrations of main ambient air pollutants such as NO_2_, PM_10 _and SO_2 _were associated with preterm births. Analysis based on the single air pollutant model indicated that cumulative effects of NO_2_, PM_10 _and SO_2 _reached the peak value on the 3^rd ^day, 4^th ^day and 3^rd ^day respectively. An increased concentration of 100 μg/m^3 ^of air pollutants NO_2_, PM_10_, SO_2 _on day 0, corresponded to RR of 1.0542 (95%CI: 1.0080 ~1.1003), 1.0688 (95%CI: 1.0074 ~1.1301), 1.1298 (95%CI: 1.0480 ~1.2116) respectively. As for the three pollutant model analysis, after adjusting the collinearity by principal component analysis, an increased concentration of 100 μg/m^3 ^led to RR values on day 0 as 1.0185 (95%CI: 1.0056~1.0313), 1.0215 (95%CI: 1.0066 ~1.0365), 1.0326 (95%CI: 1.0101 ~1.0552) for NO_2_, PM_10 _and SO_2 _respectively; RR for the strongest cumulative effects were presented as 1.0219 (95%CI:1.0053~1.0386), 1.0274 (95%CI:1.0066~1.0482), 1.0388 (95%CI: 1.0096 ~1.0681). The extent of risk resulted in this study was relatively lower than that of the research results by Sagiv [16], Liu [17], Hansen [18], Maroziene [19], Xu [20], Zhang [21] and Tsai [22]. There would be several possible causes, such as the different levels of ambient air pollution, population susceptibility, particular components and research methods, and further studies are required.

Currently the most popular were single air pollutant model involving only one single index without considering the inner link between different indexes, which could lead to in certain limitations when using the single air pollutant model. If multiple air pollutant indexes were directly fitted into the model, co linearity could inevitably confound the model due to the non-independence between different air pollutant indexes, thus leading to the instability of the model. In order to solve the problem, principal component analysis was adopted to adjust the co linearity of different air pollutant indexes in the multiple air pollutants model. Principal Component analysis is a multivariate statistical analysis method that combines three air pollutant indexes by means of an appropriate linear model, and then it generated an independent and specific composite latent variable (Principal Component) with extracted variation information of original index to establish equation of linear regression of the logarithmic latent variable and dependent variable, and as a result, the latent variable was converted into the original independent variable. By doing this, not only did the evaluation of the composite effects of different air pollutants become easier, but also the issue of co linearity among multiple air pollutants was resolved.

Mechanisms of the effect of air pollution on prematurity remain unclear. Recent studies indicated preterm delivery may be caused by inflammatory reactions, immune reactions and endocrine adjustments. Some suggested that air pollution could activate the fetal hypothalamic-pituitary-adrenal axis (HPAA), which can stimulate uterine contractions and premature rupture of fetal membranes and consequently cause preterm birth [40]. Study conducted by Peters [41] and other researchers in 1997 suggested that exposure to PM_10 _and SO_2 _during late pregnancy could cause inflammation, thus changing the blood viscosity and cause preterm birth. Knotternus [42] and others also found that inflammation could also affect placenta hypoperfusion and as a result induce preterm birth.

In order to quantitatively evaluate the interactions between air pollution and preterm birth, a time-series Generalized Additive Model (GAM) extended Poisson regression was applied. It has been widely applied in evaluating the health effect of air pollution. Since the relationship between air pollution and preterm birth could be confounded by time related variables, it is difficult to allow for evaluation when applying simple linear relationship model. This study was conducted with non-parametric smooth function to control the confounding factors such as time trends, season and weather, which provides a more powerful way of evaluating the relationship than traditional methods.

Sensitivity testing of the model suggests that due to the co linearity, point estimate of each pollutant index in the multiple air pollutants model generally went down in terms of risk effects and showed no statistical significance and instability. Comparison analysis between lag effects and cumulative effects indicated a short-term effect for the former, therefore, cumulative effects was more sensitive than lag effects. Given this observation, time-series Generalized Additive Model (GAM) extended Poisson regression combined with Principal Component regression analysis was conducted in the study. This model could not only control the confounding factors such as long-time trends, season and meteorological factors, but also resolve the co linearity among different air pollutants. In addition, effects on day 0 of multiple air pollutants and the strongest cumulative effects model were also incorporated so as to evaluate the health effects of various air pollutants on preterm birth in a comprehensive manner.

The limitation of this study was that outdoor air pollution data were from fixed monitoring locations. It might underestimate the impact of air pollution when an air pollution monitoring data was used to represent individual exposure level [43]. In addition, the time span of this study was only one year which might not be long enough to see all effects. The data were not analyzed on season and age groups, so it did not fully take into account the effect of seasonal variations as well as age difference of susceptibilities toward air pollution. Another limitation of this study was that we only studied NO_2_, PM_10_, SO_2_, by not the impact of the other pollutants as CO and O_3_. Because of the correlations between the pollutants, we cannot conclude that the preterm were caused by the three pollutants in our study or rule out the possibility of some other deleterious air pollutants.

Despite the limitations of research data, the result of this study indicated positive effects of the NO_2_, PM_10 _and SO_2 _on preterm birth risk. Although the absolute increase of risk is relatively small, we still need to take into accounts that the air pollution is a long term public health challenge, as everyday and everyone is being exposed to it, especially the pregnant women, thousands of pregnant women could have been exposed to high levels of air pollution in a long-term period. Therefore, the public health significance can not be ignored. Studies regarding the impact of air pollution on preterm deliveries are still rare in China. This study explored the potential exposure-reaction between preterm birth and air pollutants such as NO_2_, PM_10 _and SO_2 _and aimed to provide scientific tools and facts to help relevant departments in their decision-making regarding air pollution control, and we also expect more studies in the upcoming years which could be inspired by our study and in the long run could help reduce the adverse maternal outcomes.

## Conclusions

In summary, this paper has examined that the concentrations of the NO_2_, PM_10 _and SO_2 _of air pollutants contributed to occurrence of preterm birth in Guangzhou city, and has shown that the three air pollutants have dose-response reactions in terms of neonatal prematurity, through analyzing a single air pollutant model and a multiple air pollutants GAM model. Although there were limitations in this study, it provided the fact that air pollution plays a non-neglectable role in prematurity. Thus, it highlights the importance of policy-makers making decisions to control air pollution and decrease rate of preterm birth.

## Abbreviations

CI: confidence interval; DOW: day of the week; GAM: Generalized Additive Model; GCV: Generalized cross-validation; HPAA: hypothalamic-pituitary-adrenal axis; LMP: last menstrual period; NO_2_: nitrogen dioxide; PM_10_: particulate matter less than 10 μm in aerodynamic diameter; RR: relative risk; SD: standard deviation; SO_2_: sulfur dioxide.

## Competing interests

The Authors declare that they have no competing interests.

## Authors' contributions

QGZ and YKD were involved in the development of the study's protocol design, data collection, data quality monitoring, data analysis and preparation of the manuscript. JZ and SJT were involved in analysis and editing draft manuscripts. ZJL were involved mainly in data analysis and its data quality management. All authors contributed to the revision of the final manuscript.
